# Characterization of T-cell subsets in response to foot-and-mouth disease bivalent inactivated vaccine in Chinese Holstein cows

**DOI:** 10.1128/spectrum.01029-23

**Published:** 2023-10-10

**Authors:** Zhipeng Zhang, Dasheng Wang, Yiyang Yao, Jiayu Yang, Zhangping Yang, Yi Yang

**Affiliations:** 1 College of Animal Science and Technology, Yangzhou University, Yangzhou, China; 2 Jiangsu Co-innovation Center for Prevention and Control of Important Animal Infectious Diseases and Zoonoses, College of Veterinary Medicine, Yangzhou University, Yangzhou, China; University of Prince Edward Island, Charlottestown, Prince Edward Island, Canada

**Keywords:** cow, foot-and-mouth disease, vaccine efficacy, T-cell response, flow cytometry

## Abstract

Traditional vaccine efficacy evaluation predominantly relies on antibody levels, while the assessment of T-cell responses remains underexplored. In this study, we employed multi-parameter flow cytometry to comprehensively analyze T-cell responses in cows vaccinated against foot-and-mouth disease (FMD). We categorized the cows into high and low vaccine potency groups based on antibody levels and investigated differences in T-cell responses between these groups. Phenotypic analysis revealed a significant reduction in CD4, CD8, and γδ T cells in peripheral blood following FMD vaccine inoculation, concomitant with altered CD44 expression. Intriguingly, FMD vaccination induced a marked increase in the percentage of CD4^+^CD8^+^ double-positive (DP) T cells in cow peripheral blood. Notably, cows with high vaccine potency exhibited a significantly higher proportion of CD4^+^CD8^+^ DP T cells compared to those with low potency post-vaccination, suggesting their potential involvement in FMD vaccine-induced immune protection, possibly through the regulation of B-cell antibody secretion. Additionally, FMD vaccination led to the generation of central memory CD4 and CD8 T cells but not γδ T cells. Our groundbreaking findings shed light on the latent function of CD4^+^CD8^+^ DP T cells in FMD vaccine-induced immune protection and established a novel method for evaluating FMD vaccine efficacy based on T-cell responses.

**IMPORTANCE:**

Vaccination plays a crucial role in the prevention and control of FMD; however, outbreaks persist occurring worldwide. Assessing the immune response to FMD vaccines is essential for effective prevention of FMD. In this study, a seven-color flow cytometry protocol was developed to systematically evaluate the T-cell response of Chinese Holstein cows vaccinated with FMD bivalent inactivated vaccine. Our findings showed that while most T-cell subsets (%) decreased post-vaccination, a significant increase was observed in CD4^+^CD8^+^ DP T cells, which was consistent with the levels of specific foot-and-mouth disease virus (FMDV) antibodies. These findings suggested that CD4^+^CD8^+^ DP T cells could serve as a potential biomarker for the evaluation of cellular and humoral responses to FMDV vaccination. Additionally, we should be aware of the potential decline in cellular immunity among cattle during FMD vaccination, as this may increase the risk of other pathogen-related issues.

## INTRODUCTION

Foot-and-mouth disease (FMD) is a severe and highly infectious disease caused by the foot-and-mouth disease virus (FMDV). It is characterized by fever, blistering, and ulceration of oral mucosa and hoof (1). There are seven serotypes of foot-and-mouth disease virus, including A, C, O, Southern African Territories SAT 1, SAT 2, SAT 3, and Asia 1 (2). FMD inflicts significant economic losses on dairy farms worldwide, impacting milk yield and reproductive performance in dairy cows (3, 4). Vaccination serves as the primary means of controlling FMD transmission in dairy cows. However, the protection conferred by FMD vaccines is relatively short-lived and may vary, depending on the strain, but it can be improved and extended by optimizing vaccine efficacy and administering booster doses (1). Therefore, it is crucial for veterinarians to comprehend the efficacy of FMD vaccines and fine-tune the dosing and immunization intervals.

Various methods are available for evaluating the effectiveness of FMD vaccines. Conventionally, prior to registering a new vaccine strain, the vaccine’s efficacy is assessed through the inoculation of cattle’s tongues with the virulent (homologous) FMDV as the vaccine strain, followed by monitoring for 21–28 days. If the challenge virus is prevented from spreading to the feet and causing blisters, cows are considered as protected (1). However, this method is laborious and expensive for routine use. While challenge tests cannot be replaced entirely, there is a need for alternative evaluation methods. The immune response to FMDV is characterized by the induction of lymphocyte stimulation responses and the production of serotype-specific neutralizing antibodies (5). Antibody levels, commonly measured using virus neutralization tests or indirect enzyme-linked immunosorbent assays (ELISA) (6, 7), are widely employed as predictors of protection against FMD in vaccinated animals. Nevertheless, few studies have explored the T-cell responses induced by FMD vaccines in cows (8, 9), despite the potential of this approach as a novel means of evaluating vaccine effectiveness.

In China, FMD has caused substantial economic losses to dairy farms, with serotypes O and A being endemic in many provinces of northwest and southeast China since 2010 (10). The bivalent FMD vaccine has effectively controlled the spread of FMDV in China. However, The recent outbreak of FMD in developing countries has served as a wake-up call (11, 12), reminding us of the mutating nature of viruses and the possibility of a more threatening FMDV subtype emerging in the future. While the neutralizing antibody levels against FMD have been extensively studied and nearly established, animals with low levels or with absence of neutralizing antibodies may still be protected when challenged with FMDV (13). Consequently, a more comprehensive understanding of the animal immune responses induced by FMD vaccines is imperative for developing and reserving additional techniques and methods for evaluation of FMD vaccine. This study evaluates the dynamic changes in T-cell subsets in the peripheral blood of cows following inoculation with bivalent inactivated FMD vaccine (serotypes O and A) and compares the differences in T-cell activation and effects in Chinese Holstein cows that responded to the bivalent vaccine with varying levels of antibody response. Our goal is to establish a method for evaluating bivalent inactivated FMD vaccine efficacy based on T-cell responses.

## MATERIALS AND METHODS

### Animal selection and vaccination

In this experiment, 210 healthy cows were carefully selected from 1,675 lactating Chinese Holstein cows from a well-managed pasture in Jiangsu Province, China, with similar birth dates, body weights, milk yields, and somatic cell scores. None of the selected cows had a history of revelation to the FMD virus and vaccine. Each selected cow was given 2 mL of the vaccine according to the vaccine instructions. During the experiment, all the cows were placed in the same room. The vaccine used in this study was traditional and commercial water in oil, bivalent inactivated vaccine (O_2_ + AKT-III strain) (Tiankangbio, Xinjiang, China; http://www.tcsw.com.cn/html/html/pc/prodetail.html?id=66&t). Peripheral blood of cows was collected from vacuum blood collection vessels containing EDTA anticoagulant by jugular vein sampling at 0, 7, and 14 days post-immunization (dpi). Peripheral blood mononuclear cells (PBMC) and plasma samples were isolated from the collected blood. These plasma samples were used to detect virus-specific antibodies.

### Analysis of bivalent inactivated FMD vaccine antigens

In the inactivated FMD vaccines, intact virion are important immune antigens, and their content, integrity, and stability determine the immune effect of the vaccine (14). The antigen of the bivalent inactivated FMD vaccine used in this study was analyzed by negative stain electron microscopy (Hitachi, Tokyo, Japan). Purified bivalent inactivated FMD vaccine samples was added to the glow-discharged, Formvar/carbon-coated copper grids to precipitated for 1 minute, and the float was absorbed by filter paper. Then uranium dioxyacetate drops were added to the copper grids to precipitate for 1 minute, and the filter paper absorbed the float. After drying at normal temperature, the images were detected by scanning projection electron microscopy at 80 kv.

### Antibody detection and phenotypic classification

The plasma samples collected from vaccinated cows at 0, 7, and 14 dpi were assayed using commercial bovine FMDV-A-Ab and FMDV-O-Ab ELISA Kits (Qiaodu, Shanghai, China) to determine the specific antibody concentrations against the bivalent inactivated FMD vaccine. The optical density (OD) values of the samples were read at 450 nm using an ELISA plate reader (Tecan, Shanghai, China), and the OD values were corrected according to a previous method (15).

Previous studies have shown that antibody-mediated immune responses are characterized by quantitative characteristics that can be phenotypically classified (16). Therefore, we used previous methods to rank vaccine effectiveness that was assessed using antibody response levels. Briefly, the corrected OD values of all antibodies were logarithmically processed. The values on days 7 and 14 after vaccination were subtracted from the values before vaccination to obtain the antibody response level, reflecting the vaccine’s efficacy. The first and third quartiles of the antibody response values were used as critical values to categorize cows into high response (HR) and low response (LR) groups for both serotype O and A specificities. Blood samples from cows in the HR (*n* = 15) and LR (*n* = 15) groups were used for the subsequent isolation of PBMCs.

### Isolation of PBMC

Density gradient centrifugation was used to separate PBMC from dairy cows. In brief, the blood sample was diluted with equal-volume phosphate buffer solution (PBS). Next, the diluted blood sample is gently dripped along the wall of the tube onto an equal volume of separating liquid. After centrifugation at 650 *g* for 30 min, the second layer of cell fluid was absorbed into 10-mL PBS for washing. The cells were resuspended in 500 µL of RPMI-1640 nutritional medium (Gibco, Shanghai, China) supplemented with 10% fetal bovine serum (FBS) (Cytiva, Tauranga, New zealand) and counted using Automated Cell Counter (Invitrogen, Shanghai, China). The PBMCs were then diluted with nutritional medium containing 10% FBS to a concentration of 1 × 10^7^ viable cells/mL.

### Flow cytometry analysis

**Fig 1:**
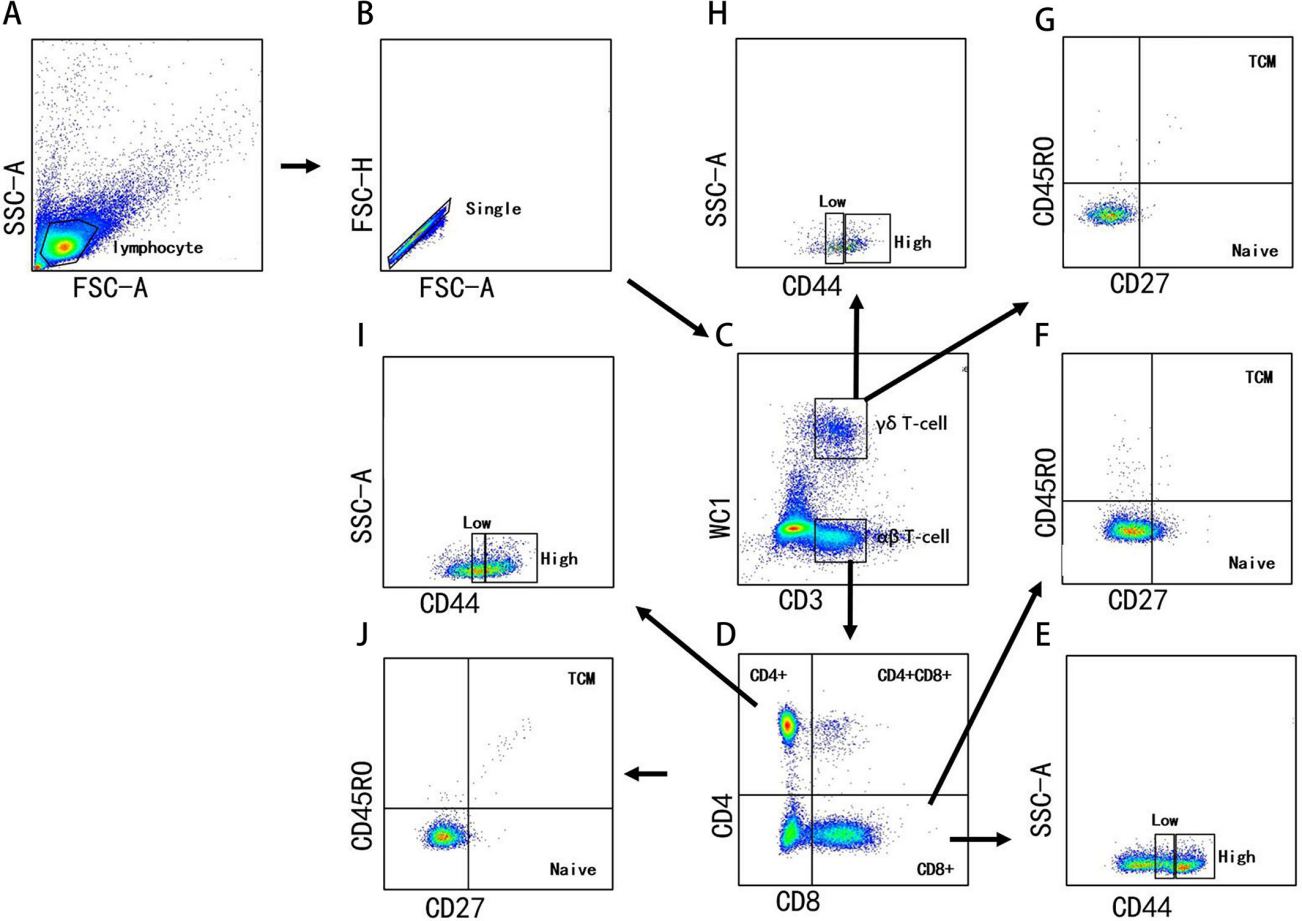
Gating strategies for phenotypic analysis of T cells in peripheral blood of Chinese Holstein cows. (**A**) Lymphocytes are isolated from all cells. (**B**) Further screening of individual cells. (**C**) The γδ (WC1^+^) and αβ (WC1^−^ CD3^+^) T cells are distinguished in lymphocytes by outputting CD3 versus WC1. (**D**) The CD4 (CD4^+^CD8^−^), CD8 (CD4^−^CD8^+^) and CD4^+^CD8^+^ double-positive T-cell subsets were defined in T cells. (**E**) The high and low subtypes were defined in activated (CD44^+^) CD8 T cells. (F) By outputting CD27 versus CD45RO, central memory T cells (Tcm) (CD27^+^CD45RO^+^) and naïve (CD27^+^CD45RO^−^) T cells are identified in CD8 T cells, respectively. (**G**) By outputting CD27 versus CD45RO, Tcm and naïve γδ T cells are identified, respectively. (**H**) The high and low subtypes were defined in activated (CD44^+^) γδ T cells. (**I**) The high and low subtypes were defined in activated (CD44^+^) CD4 T cells. (**J**) By outputting CD27 versus CD45RO, Tcm and naïve T cells are identified in CD4 T cells, respectively.

The PBMCs were plated in 96-well V-bottom plates, with 200 µL in each well. After centrifugation at 1,600 revolutions per minute for 5 min, the cells were suspended in a 50-μL mixture of antibodies with fluorescence-activated cell sorting (FACS) buffer (0.5% bovine serum albumin [BSA]-PBS). The list of antibodies is shown in Table 1. Next, the cells were stained for 20 minutes at room temperature in the dark. The cells were then washed once with FACS buffer and rotated at 400 *g* for 5 min at 4°C. At least 100,000 cells were obtained for FACS analysis. FACS LSR Fortessa (BD Biosciences, Franklin Lakes, NJ, USA) was used for flow cytometry, and FlowJo software (Tree Star Inc., Ashland, OR, USA) was used for the data analysis. The gating policy is based on fluorescence minus one and is shown in Fig. 1.

**TABLE 1:** Antibodies used for flow cytometry in this study

Marker	Clone	Isotype	Conjugate	Conjugate type	Labeling strategy
CD3	MM1A	IgG_1_	PerCP-Cy5.5	Secondary antibody	Kingfisher
WC1	CC15	IgG2a	FITC	Direct conjugate	Bio-Rad
CD4	CC8	IgG2a	Alexa Fluor 647	Direct conjugate	Bio-Rad
CD8	CC63	IgG2a	PE	Direct conjugate	Bio-Rad
CD27	M-T271	IgG1, κ	Brilliant Violet 510	Direct conjugate	BioLegend
CD44	IMC	IgG2b, κ	PrestoBlue	Direct conjugate	BioLegend
CD45RO	IL-A116	IgG3	PE/Cy7	Secondary antibody	Kingfisher

### Statistical analyses

All data were analyzed using SPSS software (IBM Corporation, Armonk, NY, USA). The normality and homogeneity of variance of the data were tested using the Kolmogorov-Smirnov test. Data are expressed as mean ± standard deviation (SD). For comparisons between two groups with normally distributed data, one-way Student *t*-tests were performed. For comparisons among multiple groups, analysis of variance tests were used. A *P* value of less than 0.05 was considered significant.

## RESULTS

### Intact structure of FMDV capsid in bivalent inactivated FMD vaccine

**Fig 2:**
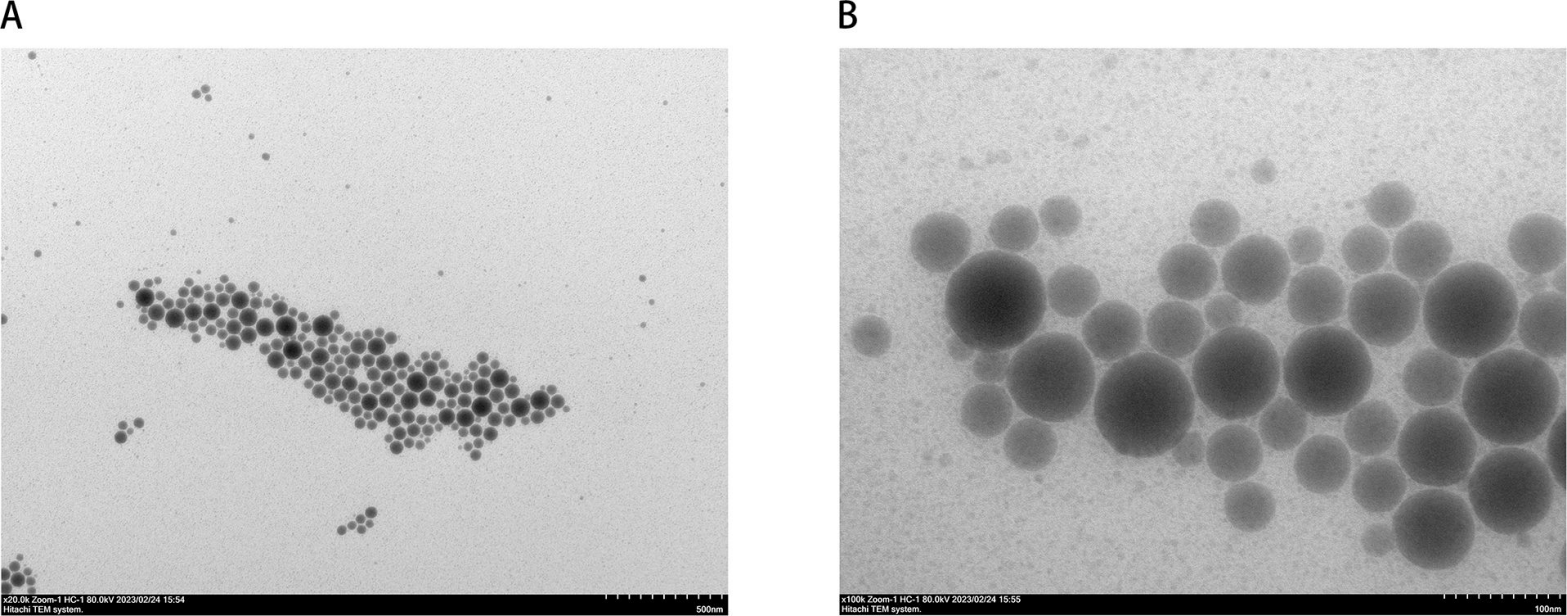
Demonstration of virus particles in vaccines. (**A**) Negative stain electron microscope (EM) images of FMD vaccine (serotypes O and A). The scale bar indicates 500 nm. (**B**) Negative stain EM images of FMD vaccine (serotypes O and A). The scale bar indicates 100 nm.

Chemical inactivation of viral particles to produce commercial vaccines may make them more unstable and can rapidly transform them into immunogenically incompetent pentamer subunits (17, 18). We observed the structure of the virus in the vaccine using negative stain electron microscopy. The results showed that many FMD virions were observed in the vaccine used in this study (Fig. 2A). The FMD virion appeared spherical; the capsid structure was complete; and the edges were smooth. No dissociated pentameric subunits were observed (Fig. 2B).

### Significant increase in plasma antibody levels after vaccination

**Fig 3:**
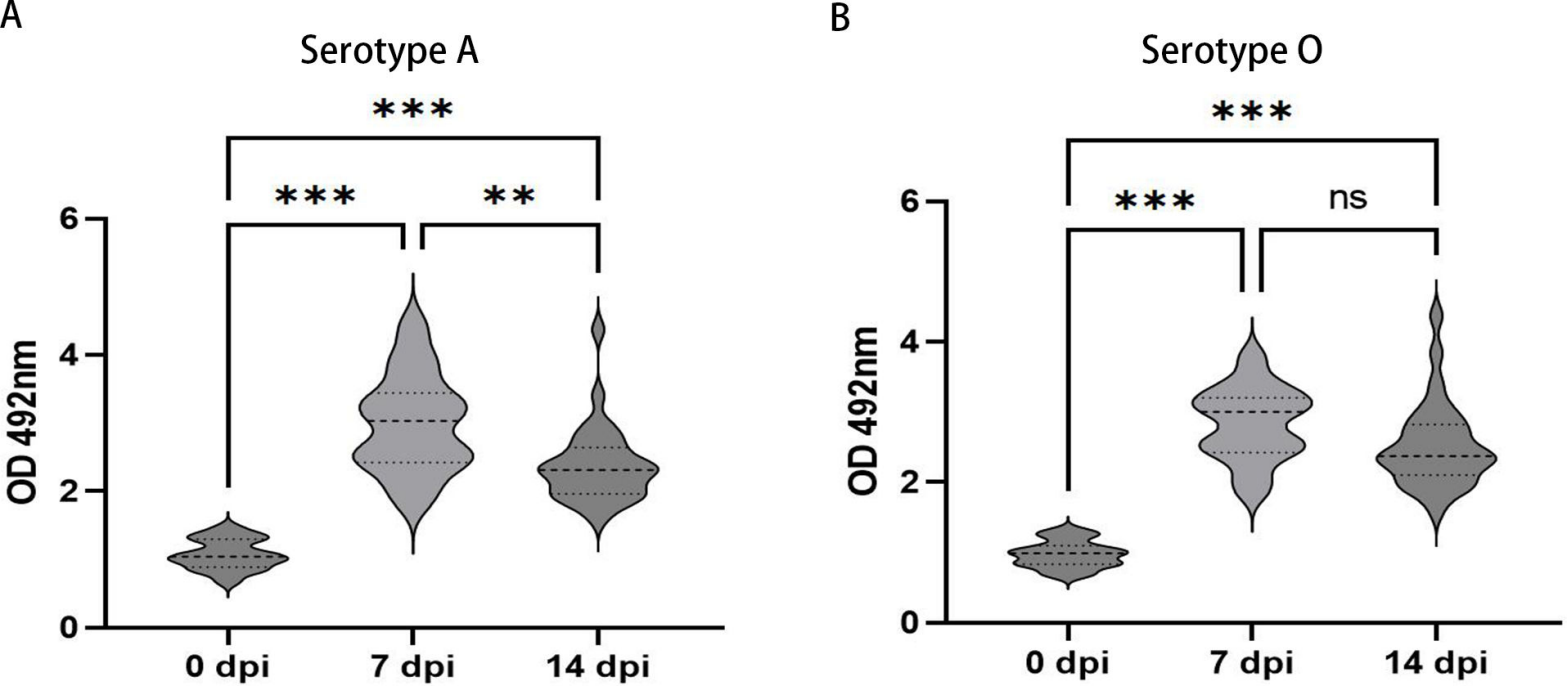
Evaluation of antibody levels of bivalent inactivated FMD vaccine in cows after inoculation. (**A**) FMD vaccine serotype A-specific antibodies were detected by ELISA. (**B**) FMD vaccine serotype O-specific antibodies were detected by ELISA. Data shown are mean ± SD from 210 cows. **P < 0.01, ***P < 0.001. ns, not significant.

To assess the efficacy of the foot-and-mouth disease vaccine used in this study and to identify potential T-cell response markers, we measured plasma antibody concentrations of cows after vaccination using ELISA. As expected, higher levels of FMDV (serotypes O and A) antibodies were detected at 7 and 14 dpi (Fig. 3A and B). Additionally, plasma FMDV (serotype A) antibodies at 14 dpi were significantly lower than those at 7 dpi but remained significantly higher than before immunization.

### Bivalent inactivated FMD vaccine induces decreased percentage of αβ and γδ T cells but not CD4^+^CD8^+^ double-positive T cells

**Fig 4:**
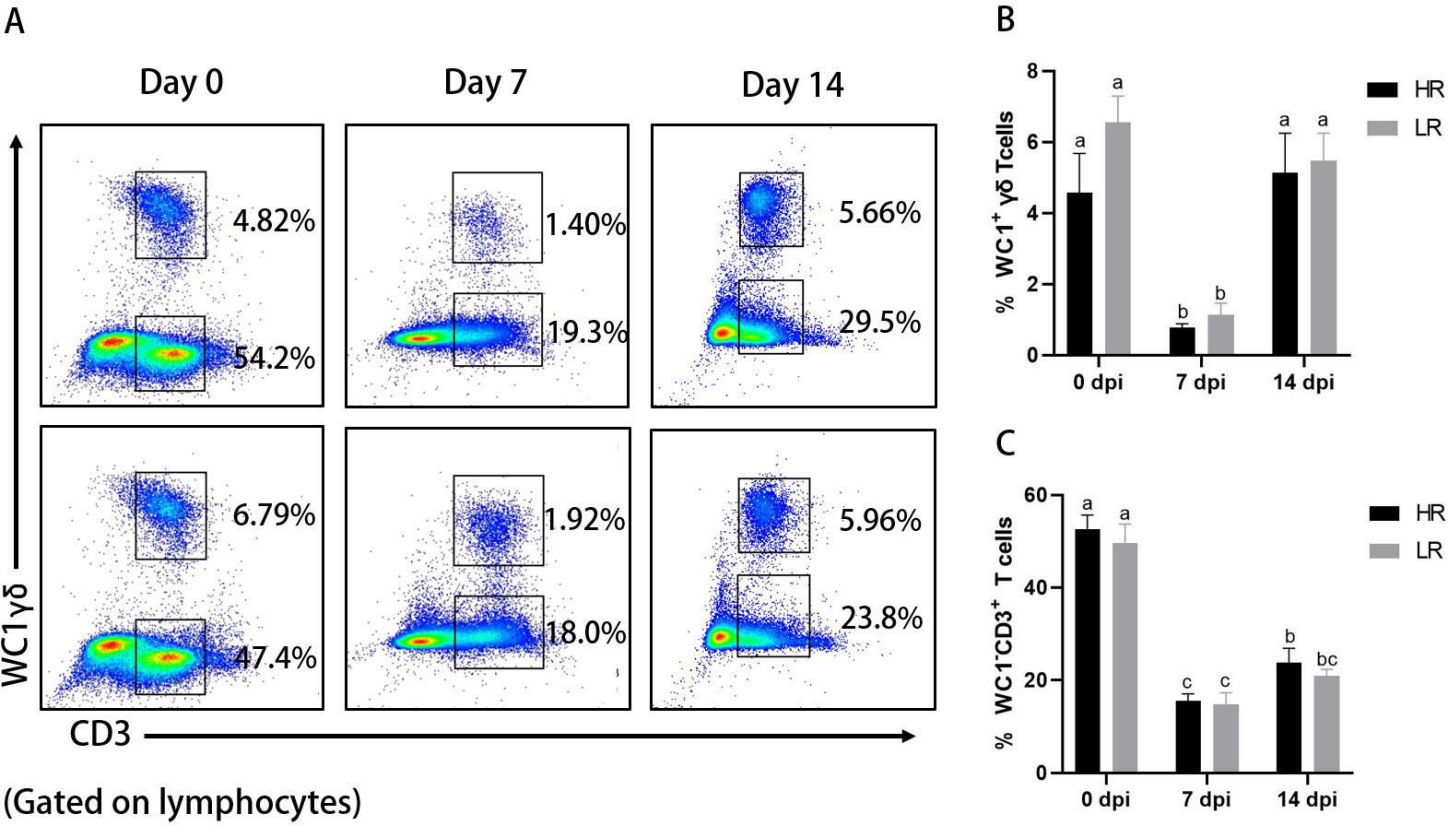
Dynamic changes in cow αβ and γδ T cells after FMD vaccination. (**A**) Representative dot plots depict γδ T cells and αβ (WC1^−^CD3^+^) T cells in the peripheral blood of indicated cows at 0, 7, and 14 dpi. Numbers indicate the percentages of γδ T and CD3 T cells. (**B**) Dynamic changes in γδ T-cell percentage in lymphocytes after FMD vaccination. (**C**) Dynamic changes in αβ T-cell percentage in lymphocytes after FMD vaccination. Data shown are mean ± SD (*n* = 15). Different letters represent significant differences between groups (*P* < 0.05). HR, high antibody response against both serotypes O and A; LR, low antibody response against both serotypes O and A.

FMD is characterized by significant lymphocytopenia associated with immunosuppression (19). However, the dynamic changes in different T-cell subsets in peripheral blood of cows after inoculation with bivalent inactivated FMD vaccine have not been well characterized. To address this, we employed a seven-color flow cytometry panel to characterize T-cell immune responses in cows following FMD vaccination. As shown in Fig. 4, the percentages of αβ (WC1^−^CD3^+^) and γδ (WC1^+^ CD3^+^) T cells in the peripheral blood of cows at 7 dpi with bivalent inactivated FMD vaccine were significantly reduced compared to those before vaccination. These reductions were recovered at 14 dpi, where the percentage of γδ T cells had fully returned to pre-vaccination levels, while αβ T cells may take longer. These changes did not significantly differ between the HR and LR groups, suggesting that these indicators may not replace existing methods of evaluating vaccines using antibody levels.

**Fig 5:**
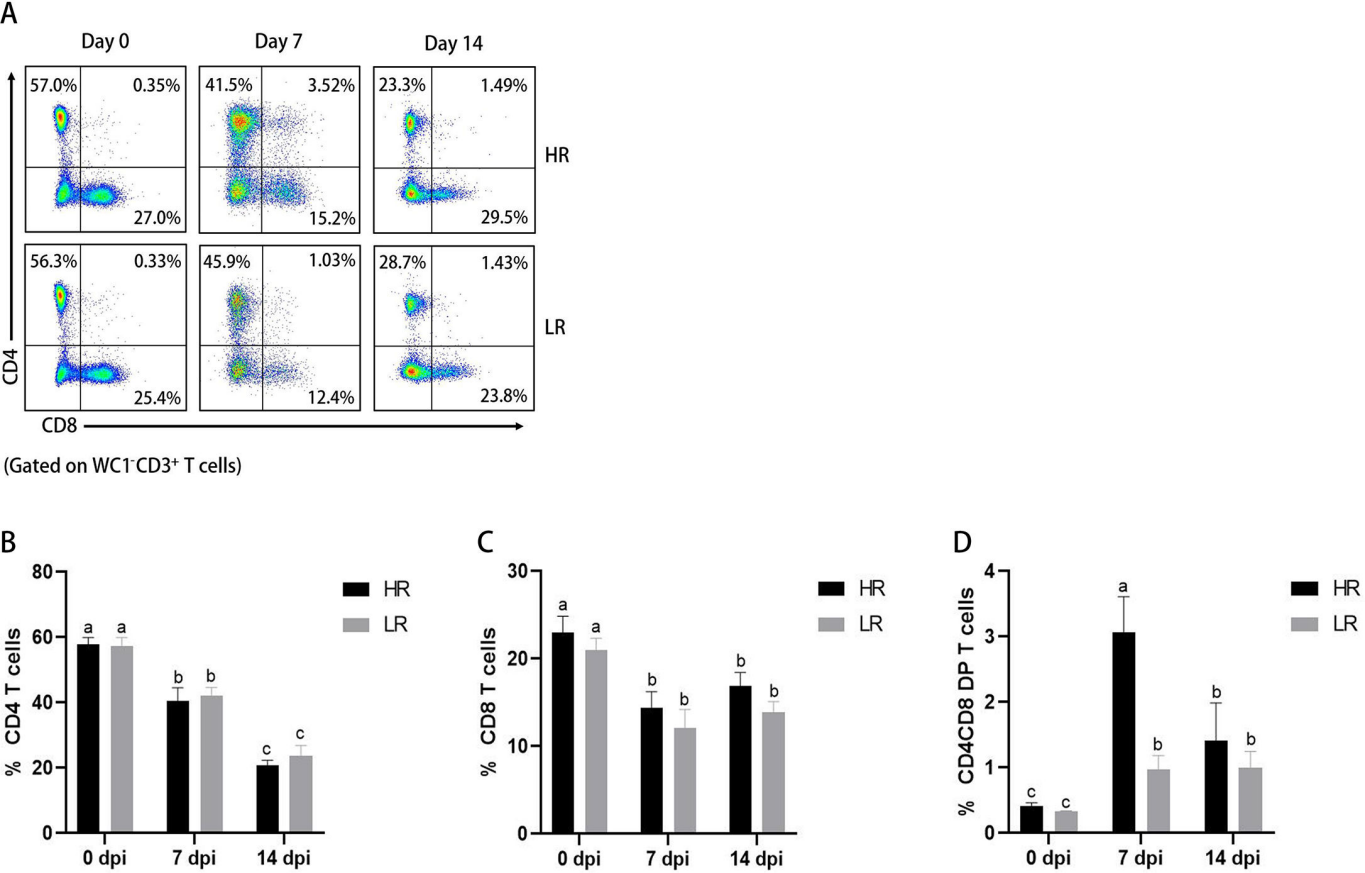
Dynamic changes in cow CD4, CD8, and DP T cells after FMD vaccination. (**A**) Representative dot plots depict CD4, CD8, and DP T cells in the peripheral bloods of indicated cows at 0, 7, and 14 dpi. Numbers indicate the percentages of CD4, CD8, and DP T cells. (**B**) Dynamic changes in CD4 T-cell percentage in αβ T cells after FMD vaccination. (**C**) Dynamic changes in CD8 T-cell percentage in αβ T cells after FMD vaccination. (**D**) Dynamic changes in CD4CD8 DP T-cell percentage in αβ T cells after FMD vaccination. Data shown are mean ± SD (*n* = 15). Different letters represent significant differences between groups (*P* < 0.05). HR, high antibody response against both serotypes O and A; LR, low antibody response against both serotypes O and A.

Furthermore, it remained unclear whether the dynamics of different T-cell subsets differed between the two groups. Therefore, we distinguished three T-cell subsets (CD8, CD4, and CD4CD8 double-positive) in the WC1γδ^−^CD3^+^ T cells (Fig. 5A). As illustrated in Fig. 5B and C, FMD vaccination elicited significantly lower percentages of CD4 and CD8 T cells in the two groups at 7 and 14 dpi. Intriguingly, the percentage of CD4^+^CD8^+^ T cells in the HR and LR groups increased significantly to varying degrees after inoculation with bivalent inactivated FMD vaccine (Fig. 5D). The percentage of CD4^+^CD8^+^ double-positive (DP) T cells in the HR group was significantly higher than that in the LR group at 7 dpi. At 14 dpi, the percentage of CD4^+^CD8^+^ DP T cells remained significantly higher than the pre-vaccination level. This suggests that the increase in the percentage of CD4^+^CD8^+^ DP T cells in the peripheral blood of cows after vaccination may serve as a signal of the efficacy of the bivalent inactivated FMD vaccine.

### Bivalent inactivated FMD vaccine induces differential activation of CD4, CD8, and γδ T cells in cows

**Fig 6:**
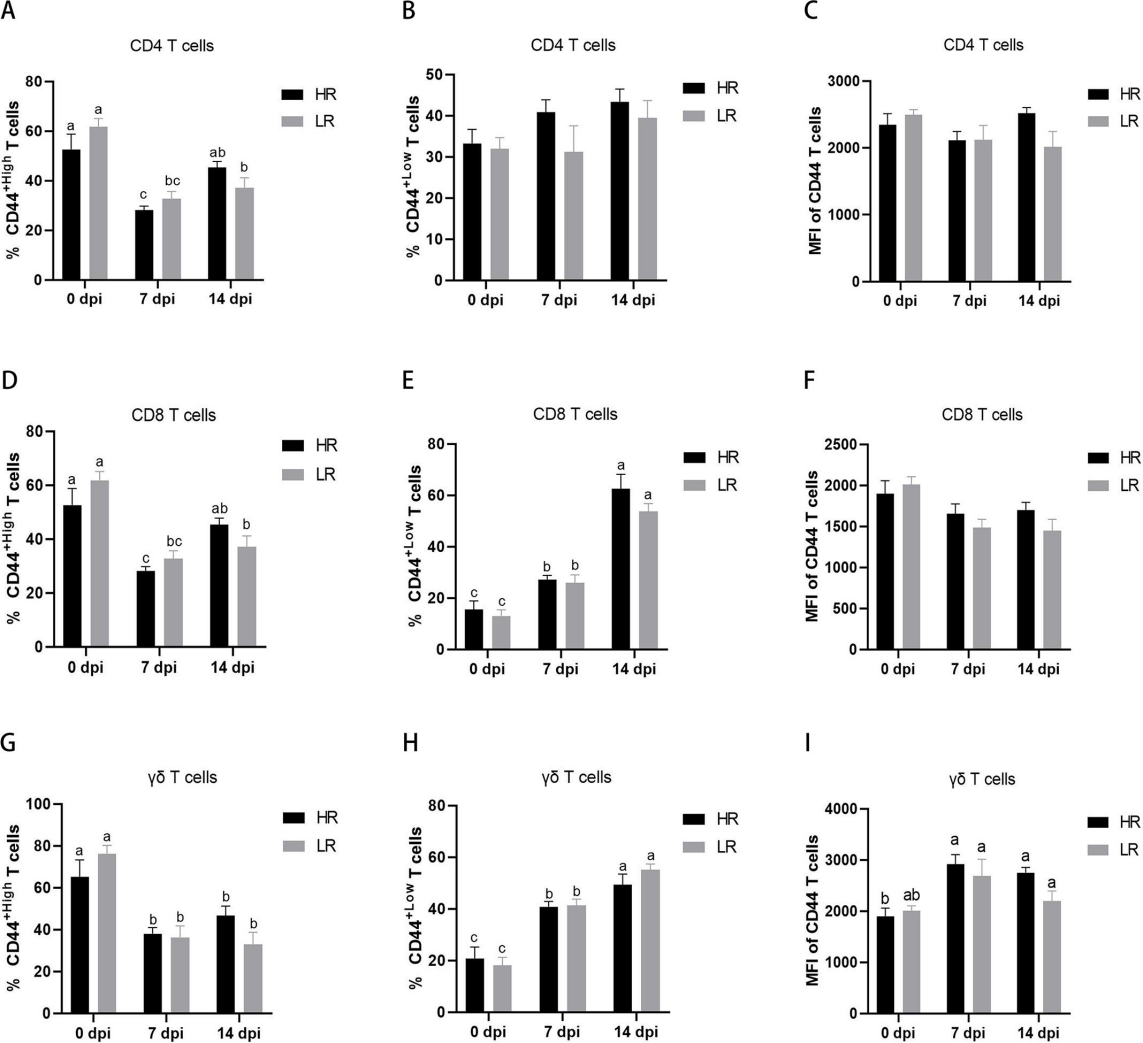
Differences in the expressions of CD44 in different T-cell subsets (CD4, CD8, and γδ) of HR and LR groups. The percentages of CD44 high (**A**) or low (**B**) expressing cells and MFI (**C**) were compared in CD4 T cells in blood between HR and LR cows. The percentages of CD44 high (**D**) or low (**E**) expressing cells and MFI (**F**) were compared in CD8 T cells in blood between HR and LR cows. The percentages of CD44 high (**G**) or low (**H**) expressing cells and MFI (**I**) were compared in γδ T cells in blood between HR and LR cows. Data shown are mean ± SD (*n* = 15). Different letters represent significant differences between groups (*P* < 0.05). HR, high antibody response against both serotypes O and A; LR, low antibody response against both serotypes O and A.

To determine the activated phenotype of T cells in vaccinated cows, vaccine-experienced T cells were identified based on high or low expression of CD44. Phenotypic analysis of CD4 T cells revealed a reduced percentage of the CD44^+high^ phenotype in T cells from cows vaccinated with the bivalent inactivated FMD vaccine (Fig. 6A). The percentage of the CD44^+low^ phenotype and the mean fluorescence intensity (MFI) of CD44 in CD4 T cells remained unchanged (Fig. 6B and C). Following FMD vaccination, CD8 T cells exhibited a reduced percentage of the CD44^+high^ phenotype and an increased percentage of the CD44^+low^ phenotype (Fig. 6D and E). The MFI of CD44 in CD8 T cells showed no significant difference before and after vaccination (Fig. 6F). Similar phenotypic changes of CD44^+high^ or CD44^+low^ were observed in γδ T cells and CD8 T cells before and after vaccination (Fig. 6G through I).

### Significant up-regulation of memory T cells at 7 dpi

**Fig 7:**
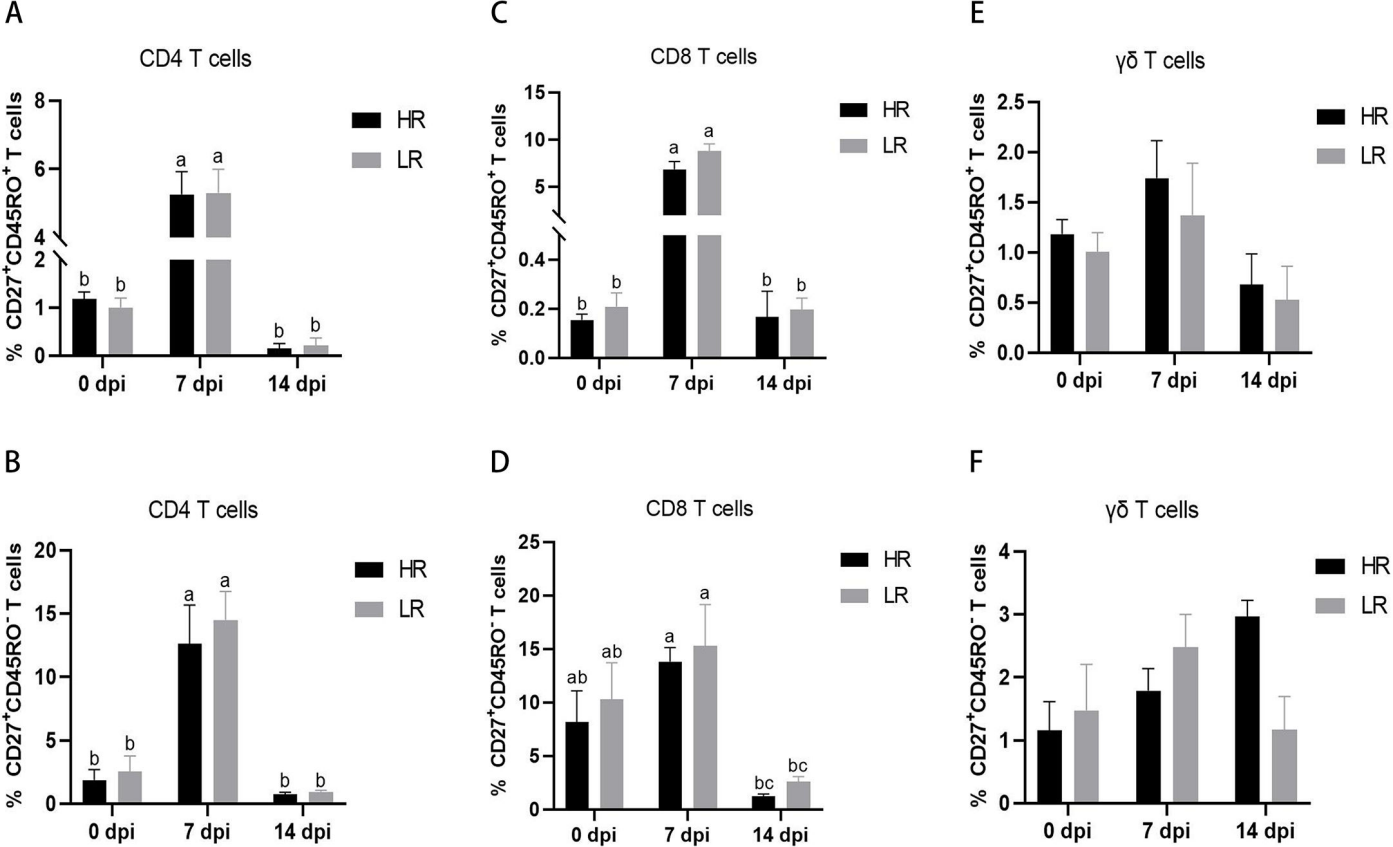
Dynamic changes in cow memory T cells after FMD vaccination. The percentages of CD27^+^CD45RO^+^ T cells (Tcm) were compared in CD4 (**A**), CD8 (**C**), and γδ (**E**) T cells in whole blood between HR and LR cows. The percentages of CD27^+^CD45RO^−^ T cells (naïve) were compared in CD4 (**B**), CD8 (**D**), and γδ (**F**) T cells in whole blood between HR and LR cows. HR, high antibody response against both serotypes O and A; LR, low antibody response against both serotypes O and A.

The expression of cell surface memory markers CD45RO and CD27 is associated with functional segregation of T-cell memory subsets in Holstein cows. CD27, a costimulatory molecule, has been normally used to identify the stages of T-cell differentiation (20). Normally, bovine memory T cells are identified based on CD45RO expression (21). The naïve cells are defined as CD45RO^−^CD27^+^, and central memory T cells are defined as CD45RO^+^CD27^+^ in this study. As proven in Fig. 7, the percentage of central memory T cells in CD4 and CD8 T cells increased significantly at 7 days after vaccination compared with pre-vaccination and returned to pre-immunization levels at 14 days. However, this difference was not significant in γδ T cells. The dynamic changes in naïve phenotype cells in CD4, CD8, and γδ T cells were different. In CD4 T cells, the percentage of naïve T cells was significantly up-regulated at 7 dpi and returned to the pre-immunization level at 14 dpi. In CD8 T cells, the percentage of naïve T cells was not significantly increased at 7 dpi but significantly decreased at 14 dpi. There existed no crucial distinction in the percentage of γδ T cells with naïve phenotype before and after vaccination.

## DISCUSSION

Previous studies evaluating vaccine efficacy have commonly focused on antibody levels as a measure, overlooking the significance of T cell-mediated cellular immunity (22). However, robust T-cell responses are vital for pathogen clearance and the formation of strong memory responses (23). In this research, we utilized a seven-color flow cytometry method to evaluate cellular immunity in dairy cows, specifically the T-cell immune response to the bivalent inactivated FMD vaccine. By doing so, we aimed to establish a standardized method for evaluating the efficacy of bivalent inactivated FMD vaccine based on T-cell responses. While a previous study had established a cattle T-cell phenotyping method using an eight-color panel, the function of identified cell subsets in vaccinated or infected cattle had not been determined (24). In contrast, the cell subset functions identified in our study were well established, making them more reliable for the evaluation of T-cell responses in dairy cows. In this study, we did not set up virus-infected cows as controls. On the one hand, it is too costly to establish an adequately powered parallel group trial of FMDV infection. On the other hand, the level of virus-specific antibodies is sufficient to determine vaccine immunogenicity, which is widely accepted by researchers and farmers (22, 25). In addition, a large number of viral particles with intact capsid structure were observed by negative staining scanning electron microscopy. This result further assured the immunogenicity of the vaccine.

Before assessing the T-cell immune response to bivalent inactivated FMD vaccine in dairy cows, we detected virus-specific antibodies by ELISA tests; the result showed that a significant increase in antibody concentrations was detected in the plasma of Chinese Holstein cows at 7 dpi. Although antibody concentrations decreased at 14 dpi, they remained above pre-immunization levels. In our study, OD value and change regularity of antibody of vaccinated cows were compatible with earlier research (26, 27). The FMD vaccine shows different efficacies in different individual cows (28). Therefore, we used the level of vaccine-specific antibodies produced by individuals to classify cows into high- and low-response groups, which represent high vaccine efficacy and low vaccine efficacy in cows, respectively. Since antibody-mediated immune response and cell-mediated immune response are mutually regulated (29), this grouping design may help us screen out T-cell response indicators that can take into account antibody-level factors to evaluate vaccine efficacy. This clustering strategy has been used in dairy cattle breeding efforts for disease resistance (30). Previous studies have assessed the possibility of phenotypic classification of immune responses in cows using mean ± SD and quartiles (31, 32). In this study, ranking responses were divided into HR and LR using quartiles, which reduced the variability in the number of cows classified as HR or LR due to means and SD differences and allowed comparison of phenotypic with genetic rankings.

Based on the above grouping of cows, we characterized the dynamic changes in different T-cell subsets in the peripheral blood of cows after vaccination with inactivated FMDV. We noted that the percentage of CD4, CD8, and γδ T cells in the peripheral blood of cows after FMDV vaccination was significantly reduced and did not fully recover 14 days after vaccination. Peripheral blood lymphocytopenia, or lymphocytopenia, after FMDV infection is a common feature in pigs (33), cattle (34), and mice (35). Lymphopenia is considered to be one of the important mechanisms by which FMDV evades the host immune response and induces immune suppression (36). A previous research on cattle indicated that FMDV down-regulates CD4, CD8, and γδ T cells up to 48 h post-immunization (37). By contrast, Garcia-Valcarcel et al. recorded no significant difference in the numbers of circulating CD4 T cells in the first vaccinated/infected bovines (38). One possible reason is that the vaccine used in that study was not the inactivated virus but the capsid protein VP1. Our study is the first to report that an bivalent inactivated FMD vaccine causes T-cell depletion in dairy cows lasting up to 14 days. Lymphopenia may delay pathogen clearance in favor of macrophage stimulation (39) and the accompanying “cytokine storm” that leads to host organ dysfunction (40) in previous cases of other viral infections (SARS-CoV-2, COVID-19, and HIV). These injuries may increase the risk of infections by other pathogens. This suggests to us that cows may have a higher risk of disease for at least 14 days after FMD vaccination and require additional special care. In our current results, we found no distinguished distinctions between the HR and LR groups, and changes in these T-cell subsets may not be directly related to B cell-mediated antibody production.

Surprisingly, we observed a significant up-regulation of CD4^+^CD8^+^ DP T cells in the peripheral blood of cows after vaccination with the bivalent inactivated FMD vaccine. CD4^+^CD8^+^ DP T cells were previously considered a developmental stage of T cells in the thymus, and it was uncommon for mature CD4^+^CD8^+^ DP T cells to be present in peripheral blood (41). Unexpectedly, previous studies in the past decades have shown the presence of mature CD4^+^CD8^+^ DP T cells in peripheral blood of humans (42), dogs (43), and pigs (44). Earlier studies have indicated that it is primarily CD4^+^CD8^+^ DP T cells that are involved in the interferon gamma responses among all subtypes of T cells in pigs (45). Besides, in porcine reproductive and respiratory syndrome virus disease, CD4^+^CD8^+^ DP T cells appear to have a certain cytotoxic function (46). The CD4^+^CD8^+^ DP T cells make up a enormous percentage of T lymphocytes in pigs (47), in stark contrast to cows, where the function of CD4^+^CD8^+^ DP T cells in viral immunity has not been studied. Strikingly, The percentage of CD4^+^CD8^+^ DP T cells in αβ T cells of cows in the HR group was distinguishably higher than that in the LR group at 7 dpi. This suggests that the mount in the proportion of CD4^+^CD8^+^ DP T cells may be connected with antibody secretion in cows induced by FMDV vaccination. CD4 helper T cells regulate the activation and differentiation of B cells into antibody-producing plasma cells (48 – 50). In recent years, a new class of IL-21-producing memory CD4 helper T cells has been identified, suggesting that there may be many more helper T cells that have not been discovered (51). This new population, known as peripheral T helper cells (Tph), has essential similarities to Tfh cells. Therefore, we speculate that the increase in CD4^+^CD8^+^ DP T cells in αβ T cells of dairy cows after inoculation with FMD vaccination might be a Tph.

The mechanisms of lymphopenia induced by FMDV are still ambiguous. One hypothesis is that the interaction of FMDV with integrin receptors alters leukocyte adhesion properties and thus lymphocyte trasport during the peak period of viremia, which might be responsible for the down-regulation of the T-cell subpopulation after FMDV infection, as it has been revealed that FMDV infects cells via integrins of αβ T cells (52). Like integrin expression, CD44 expression is up-regulated after lymphocyte action, thereby promoting their movement in the extracellular matrix through interactions with hyaluronic acid and fibulectin (53). In this research, the expression of CD44 in CD4, CD8, and γδ T cells was detected. The results showed that the percentage of CD4, CD8, and γδ T cells expressing CD44 decreased significantly after inoculation with FMDV vaccine, and their changes were consistent with those of CD4, CD8, and γδ T cells. Furthermore, we found no distinguished distinctions in the MFI of CD44 in CD4 and CD8 T cells before and after vaccination. The MFI of CD44 in γδ T cells of the HR group increased significantly at 7 dpi compared to before vaccination. Together, these results indicate that the percentage of CD4, CD8, and γδ T cells expressing CD44 was greater in the decreased T cells caused by FMD vaccination. Therefore, our experimental results seem to support the above hypothesis.

Memory T cells produce a rapid and powerful immune response that is a hallmark of efficient vaccination (21). Thus, the quality and quantity of induced immune memory are crucial to assessing the vaccine’s effectiveness. In this study, the naïve cells are defined as CD45RO^−^CD27^+^, and central memory T cells are defined as CD45RO^+^CD27^+^. The consequences of the current research showed that the percentage of CD45RO^+^CD27^+^ T cells in CD4 and CD8 T cells increased significantly at 7 days after vaccination compared with pre-vaccination and returned to pre-immunization levels at 14 days. Over that past decades, numerous reports on humans and cattle have challenged the classical CD45RA/RO paradigm, which defined the memory phenotype as CD45RO^+^ and CD45RA^−^ cells (54, 55). For instance, cell proliferation was not observed after stimulation of CD45RO^+^CD4^+^ and CD45RO^+^CD8^+^ T cells isolated from cattle using the homogenates of the parasites (56). In contrast, our results appear to support the original classical paradigm.

In conclusion, our study systematically evaluated the dynamic changes in T-cell subsets in Chinese Holstein cattle after FMD vaccination and compared the differences in T-cell responses among individuals with different vaccine antibody responses. We observed that the percentage of CD4, CD8, and γδ T cells in peripheral blood of dairy cows was significantly decreased after inoculation with bivalent inactivated FMD vaccine, and the expression of CD44 changed synchronously. Unexpectedly, CD4^+^CD8^+^ DP T cells were significantly up-regulated after vaccination, suggesting their potential role as a T-cell response marker for the evaluation of bivalent inactivated FMD vaccine efficacy. However, the correlation between these results and challenge/protection has not been studied, and further research with different types of FMD vaccines and cattle species is recommended to generalize the results. Overall, our study highlights the importance of considering T-cell responses in evaluating vaccine effectiveness, and our findings contribute to a better understanding of the immune responses induced by bivalent inactivated FMD vaccine in dairy cows.

## References

[1] Gubbins S , Paton DJ , Dekker A , Ludi AB , Wilsden G , Browning CFJ , Eschbaumer M , Barnabei J , Duque H , Pauszek LL , King DP . 2022. Predicting cross-protection against foot-and-mouth disease virus strains by serology after vaccination. Front Vet Sci 9:1027006. doi:10.3389/fvets.2022.1027006 36532344 PMC9751447

[2] Bazid AH , Amer HM , Nayel M , Attia M , Maklad N , Wasfy M , Abdelmegeid M , El-Sayed MM , Magouz A , Badr Y . 2023. Assessment of the potency and effectiveness of a heptavalent oil-adjuvanted (ISA 206) foot-and-mouth disease vaccine in Egypt. Arch Virol 168:62. doi:10.1007/s00705-022-05624-2 36633687 PMC9836974

[3] Singh RK , Sharma GK , Mahajan S , Dhama K , Basagoudanavar SH , Hosamani M , Sreenivasa BP , Chaicumpa W , Gupta VK , Sanyal A . 2019. Foot-and-mouth disease virus: immunobiology, advances in vaccines and vaccination strategies addressing vaccine failures-an Indian perspective. Vaccines 7:90. doi:10.3390/vaccines7030090 31426368 PMC6789522

[4] Jamal SM , Belsham GJ . 2013. Foot-and-mouth disease: past, present and future. Vet Res 44:116. doi:10.1186/1297-9716-44-116 24308718 PMC4028749

[5] Abubakar M , Manzoor S , Ahmed A . 2018. Interplay of foot and mouth disease virus with cell-mediated and humoral immunity of host. Rev Med Virol 28. doi:10.1002/rmv.1966 29282795

[6] Paton DJ , Reeve R , Capozzo AV , Ludi A . 2019. Estimating the protection afforded by foot-and-mouth disease vaccines in the laboratory. Vaccine 37:5515–5524. doi:10.1016/j.vaccine.2019.07.102 31405637

[7] Ma L , Zhang J , Chen HT , Zhou JH , Ding YZ , Liu YS . 2011. An overview on ELISA techniques for FMD. Virol J 8:419. doi:10.1186/1743-422X-8-419 21888667 PMC3180423

[8] Yang L , Liu Z , Li J , He K , Kong L , Guo R , Liu W , Gao Y , Zhong J . 2018. Association of the expression of Th Cytokines with peripheral Cd4 and Cd8 lymphocyte Subsets after vaccination with FMD vaccine in Holstein young sires. Res Vet Sci 119:79–84. doi:10.1016/j.rvsc.2018.05.017 29885549

[9] Carr BV , Lefevre EA , Windsor MA , Inghese C , Gubbins S , Prentice H , Juleff ND , Charleston B . 2013. CD4+ T-cell responses to foot-and-mouth disease virus in vaccinated cattle. J Gen Virol 94:97–107. doi:10.1099/vir.0.045732-0 23034593 PMC3542717

[10] Haoran W , Jianhua X , Maolin O , Hongyan G , Jia B , Li G , Xiang G , Hongbin W . 2021. Assessment of foot-and-mouth disease risk areas in Mainland China based spatial multi-criteria decision analysis. BMC Vet Res 17:374. doi:10.1186/s12917-021-03084-5 34872574 PMC8647368

[11] Do H , Nguyen H-T-M , Van Ha P , Kompas T , Van KD , Chu L . 2022. Estimating the transmission parameters of foot-and-mouth disease in Vietnam: a spatial-dynamic kernel-based model with outbreak and host data. Prev Vet Med 208:105773. doi:10.1016/j.prevetmed.2022.105773 36228512

[12] Chanchaidechachai T , de Jong MCM , Fischer EAJ . 2021. Spatial model of foot-and-mouth disease outbreak in an endemic area of Thailand. Prev Vet Med 195:105468. doi:10.1016/j.prevetmed.2021.105468 34428641

[13] Summerfield A , Gerber H , Schmitt R , Liniger M , Grazioli S , Brocchi E . 2022. Relationship between neutralizing and opsonizing monoclonal antibodies against foot-and-mouth disease virus. Front Vet Sci 9:1033276. doi:10.3389/fvets.2022.1033276 36311653 PMC9597200

[14] Kotecha A , Seago J , Scott K , Burman A , Loureiro S , Ren J , Porta C , Ginn HM , Jackson T , Perez-Martin E , Siebert CA , Paul G , Huiskonen JT , Jones IM , Esnouf RM , Fry EE , Maree FF , Charleston B , Stuart DI . 2015. Structure-based energetics of protein interfaces guides foot-and-mouth disease virus vaccine design. Nat Struct Mol Biol 22:788–794. doi:10.1038/nsmb.3096 26389739 PMC5985953

[15] Hernández A , Karrow N , Mallard BA . 2003. Evaluation of immune responses of cattle as a means to identify high or low responders and use of a human Microarray to differentiate gene expression. Genet Sel Evol 35:S67–81. doi:10.1186/1297-9686-35-S1-S67 12927081 PMC3231764

[16] Heriazon A , Quinton M , Miglior F , Leslie KE , Sears W , Mallard BA . 2013. Phenotypic and genetic parameters of antibody and delayed-type hypersensitivity responses of lactating Holstein cows. Vet Immunol Immunopathol 154:83–92. doi:10.1016/j.vetimm.2013.03.014 23747204

[17] Doel TR , Baccarini PJ . 1981. Thermal stability of foot-and-mouth disease virus. Arch Virol 70:21–32. doi:10.1007/BF01320790 6277281

[18] Doel TR , Chong WK . 1982. Comparative Immunogenicity of 146S, 75S and 12S particles of foot-and-mouth disease virus. Arch Virol 73:185–191. doi:10.1007/BF01314726 6293410

[19] Guo Z , Zhao Y , Zhang Z , Li Y . 2021. Interleukin-10-mediated lymphopenia caused by acute infection with foot-and-mouth disease virus in mice. Viruses 13:2358. doi:10.3390/v13122358 34960627 PMC8708299

[20] Guerra-Maupome M , Palmer MV , Waters WR , McGill JL . 2019. Characterization of γδ T cell effector/memory subsets based on CD27 and CD45R expression in response to mycobacterium bovis infection. Immunohorizons 3:208–218. doi:10.4049/immunohorizons.1900032 31356167 PMC6875775

[21] Kandel A , Li L , Hada A , Xiao Z . 2022. Differential expression of CD45Ro and CD45RA in bovine T cells. Cells 11:1844. doi:10.3390/cells11111844 35681539 PMC9180881

[22] Chathuranga WAG , Hewawaduge C , Nethmini NAN , Kim TH , Kim JH , Ahn YH , Yoon IJ , Yoo SS , Park JH , Lee JS . 2022. Efficacy of a novel multiepitope vaccine candidate against foot-and-mouth disease virus serotype O and A. Vaccines (Basel) 10:2181. doi:10.3390/vaccines10122181 36560591 PMC9786174

[23] Laidlaw BJ , Craft JE , Kaech SM . 2016. The multifaceted role of CD4(+) T cells in CD8(+) T cell memory. Nat Rev Immunol 16:102–111. doi:10.1038/nri.2015.10 26781939 PMC4860014

[24] Roos EO , Mwangi WN , Gerner W , Waters R , Hammond JA . 2023. OMIP-089: Cattle T-cell phenotyping by an 8-color panel. Cytometry A 103:279–282. doi:10.1002/cyto.a.24718 36734489

[25] Tewari A , Ambrose H , Parekh K , Inoue T , Guitian J , Nardo AD , Paton DJ , Parida S . 2021. Development and validation of confirmatory foot-and-mouth disease virus antibody elisas to identify infected animals in vaccinated populations. Viruses 13:914. doi:10.3390/v13050914 34063385 PMC8156621

[26] Eblé PL , de Bruin MGM , Bouma A , van Hemert-Kluitenberg F , Dekker A . 2006. Comparison of immune responses after intra-typic heterologous and homologous vaccination against foot-and-mouth disease virus infection in pigs. Vaccine 24:1274–1281. doi:10.1016/j.vaccine.2005.09.040 16289709

[27] Khorasani A , Madadgar O , Soleimanjahi H , Keyvanfar H , Mahravani H . 2016. Evaluation of the efficacy of a new oil-based adjuvant ISA 61 VG FMD vaccine as a potential vaccine for cattle. Iran J Vet Res 17:8–12. 27656222 PMC4898013

[28] Singanallur NB , Dekker A , Eblé PL , van Hemert-Kluitenberg F , Weerdmeester K , Horsington JJ , Vosloo W . 2021. Emergency FMD serotype o vaccines protect cattle against heterologous challenge with a variant foot-and-mouth disease virus from the O/ME-SA/Ind2001 lineage. Vaccines (Basel) 9:1110. doi:10.3390/vaccines9101110 34696216 PMC8537456

[29] Crotty S . 2011. Follicular helper CD4 T cells (TFH). Annu Rev Immunol 29:621–663. doi:10.1146/annurev-immunol-031210-101400 21314428

[30] Begley N , Buckley F , Burnside EB , Schaeffer L , Pierce K , Mallard BA . 2009. Immune responses of Holstein and Norwegian red X Holstein calves on Canadian dairy farms. J Dairy Sci 92:518–525. doi:10.3168/jds.2008-1300 19164662

[31] Heriazon A , Hamilton K , Huffman J , Wilkie BN , Sears W , Quinton M , Mallard BA . 2011. Immunoglobulin isotypes of lactating Holstein cows classified as high, average, and low Type-1 or -2 immune responders. Vet Immunol Immunopathol 144:259–269. doi:10.1016/j.vetimm.2011.08.023 21930307

[32] Husseini N , Beard SC , Hodgins DC , Barnes C , Chik E , Mallard BA . 2022. Immuno-phenotyping of Canadian beef cattle: adaptation of the high immune response methodology for utilization in beef cattle. Transl Anim Sci 6:txac006. doi:10.1093/tas/txac006 35261968 PMC8896012

[33] Díaz-San Segundo F , Salguero FJ , de Avila A , de Marco MM , Sánchez-Martín MA , Sevilla N . 2006. Selective lymphocyte depletion during the early stage of the immune response to foot-and-mouth disease virus infection in swine. J Virol 80:2369–2379. doi:10.1128/JVI.80.5.2369-2379.2006 16474143 PMC1395371

[34] Saravanan S , Umapathi V , Priyanka M , Hosamani M , Sreenivasa BP , Patel BHM , Narayanan K , Sanyal A , Basagoudanavar SH . 2020. Hematological and serum biochemical profile in cattle experimentally infected with foot-and-mouth disease virus. Vet World 13:426–432. doi:10.14202/vetworld.2020.426-432 32367945 PMC7183469

[35] Salguero FJ , Sánchez-Martín MA , Díaz-San Segundo F , de Avila A , Sevilla N . 2005. Foot-and-mouth disease virus (FMDV) causes an acute disease that can be lethal for adult laboratory mice. Virology 332:384–396. doi:10.1016/j.virol.2004.11.005 15661169

[36] Stenfeldt C , Diaz-San Segundo F , de Los Santos T , Rodriguez LL , Arzt J . 2016. The pathogenesis of foot-and-mouth disease in pigs. Front Vet Sci 3:41. doi:10.3389/fvets.2016.00041 27243028 PMC4876306

[37] Joshi G , Sharma R , Kakker NK . 2009. Phenotypic and functional characterization of T-cells and in vitro replication of FMDV serotypes in bovine lymphocytes. Vaccine 27:6656–6661. doi:10.1016/j.vaccine.2009.08.107 19751690

[38] Garcia-Valcarcel M , Doel T , Collen T , Ryan M , Parkhouse RM . 1996. Recognition of foot-and-mouth disease virus and its Capsid protein Vp1 by bovine peripheral T lymphocytes. J Gen Virol 77:727–735. doi:10.1099/0022-1317-77-4-727 8627261

[39] Pasero D , Sanna S , Liperi C , Piredda D , Branca GP , Casadio L , Simeo R , Buselli A , Rizzo D , Bussu F , Rubino S , Terragni P . 2021. A challenging complication following SARS-CoV-2 infection: a case of pulmonary mucormycosis. Infection 49:1055–1060. doi:10.1007/s15010-020-01561-x 33331988 PMC7745708

[40] Peng X , Ouyang J , Isnard S , Lin J , Fombuena B , Zhu B , Routy JP . 2020. Sharing CD4+ T cell loss: when COVID-19 and HIV collide on immune system. Front Immunol 11:596631. doi:10.3389/fimmu.2020.596631 33384690 PMC7770166

[41] Germain RN . 2002. T-cell development and the CD4-CD8 lineage decision. Nat Rev Immunol 2:309–322. doi:10.1038/nri798 12033737

[42] Desfrançois J , Moreau-Aubry A , Vignard V , Godet Y , Khammari A , Dréno B , Jotereau F , Gervois N . 2010. Double positive CD4CD8 alphabeta T cells: a new tumor-reactive population in human melanomas. PLoS One 5:e8437. doi:10.1371/journal.pone.0008437 20052413 PMC2797605

[43] von Buttlar H , Bismarck D , Alber G . 2015. Peripheral canine CD4(+)CD8(+) double-positive T cells - unique amongst others. Vet Immunol Immunopathol 168:169–175. doi:10.1016/j.vetimm.2015.09.005 26460086

[44] Maggioli MF , Lawson S , de Lima M , Joshi LR , Faccin TC , Bauermann FV , Diel DG . 2018. Adaptive immune responses following senecavirus A infection in pigs. J Virol 92:e01717-17. doi:10.1128/JVI.01717-17 29142122 PMC5774895

[45] Rodríguez-Carreño MP , López-Fuertes L , Revilla C , Ezquerra A , Alonso F , Domínguez J . 2002. Phenotypic characterization of porcine IFN-gamma-producing lymphocytes by flow cytometry. J Immunol Methods 259:171–179. doi:10.1016/s0022-1759(01)00511-7 11730852

[46] Chung CJ , Cha SH , Grimm AL , Ajithdoss D , Rzepka J , Chung G , Yu J , Davis WC , Ho CS . 2018. Pigs that recover from porcine reproduction and respiratory syndrome virus infection develop cytotoxic CD4+CD8+ AND CD4+CD8- T-cells that kill virus infected cells. PLoS One 13:e0203482. doi:10.1371/journal.pone.0203482 30188946 PMC6126854

[47] Okutani M , Tsukahara T , Kato Y , Fukuta K , Inoue R . 2018. Gene expression profiles of CD4/CD8 double-positive T cells in porcine peripheral blood. Anim Sci J 89:979–987. doi:10.1111/asj.13021 29740910

[48] Rasheed AU , Rahn HP , Sallusto F , Lipp M , Müller G . 2006. Follicular B helper T cell activity is confined to CXCR5(hi)ICOS(hi) CD4 T cells and is independent of CD57 expression. Eur J Immunol 36:1892–1903. doi:10.1002/eji.200636136 16791882

[49] Chtanova T , Tangye SG , Newton R , Frank N , Hodge MR , Rolph MS , Mackay CR . 2004. T follicular helper cells express a distinctive transcriptional profile, reflecting their role as non-Th1/Th2 effector cells that provide help for B cells. J Immunol 173:68–78. doi:10.4049/jimmunol.173.1.68 15210760

[50] Chevalier N , Jarrossay D , Ho E , Avery DT , Ma CS , Yu D , Sallusto F , Tangye SG , Mackay CR . 2011. CXCR5 expressing human central memory CD4 T cells and their relevance for humoral immune responses. J Immunol 186:5556–5568. doi:10.4049/jimmunol.1002828 21471443

[51] Rao DA , Gurish MF , Marshall JL , Slowikowski K , Fonseka CY , Liu Y , Donlin LT , Henderson LA , Wei K , Mizoguchi F , Teslovich NC , Weinblatt ME , Massarotti EM , Coblyn JS , Helfgott SM , Lee YC , Todd DJ , Bykerk VP , Goodman SM , Pernis AB , Ivashkiv LB , Karlson EW , Nigrovic PA , Filer A , Buckley CD , Lederer JA , Raychaudhuri S , Brenner MB . 2017. Pathologically expanded peripheral T helper cell subset drives B cells in rheumatoid arthritis. Nature 542:110–114. doi:10.1038/nature20810 28150777 PMC5349321

[52] Jackson T , Sheppard D , Denyer M , Blakemore W , King AM . 2000. The epithelial integrin alphavbeta6 is a receptor for foot-and-mouth disease virus. J Virol 74:4949–4956. doi:10.1128/jvi.74.11.4949-4956.2000 10799568 PMC110846

[53] Dailey MO . 1998. Expression of T lymphocyte adhesion molecules: regulation during antigen-induced T cell activation and differentiation. Crit Rev Immunol 18:153–184. doi:10.1615/critrevimmunol.v18.i3.10 9637409

[54] Denis M , Lacy-Hulbert SJ , Buddle BM , Williamson JH , Wedlock DN . 2011. Streptococcus uberis-specific T cells are present in mammary gland secretions of cows and can be activated to kill S. uberis. Vet Res Commun 35:145–156. doi:10.1007/s11259-011-9462-1 21279814

[55] Anmol K , Akanksha H , Zhengguo X . 2022. Are CD45RO+ AND CD45RA- genuine markers for bovine memory T cells Animal Diseases 2:23. doi:10.1186/s44149-022-00057-5

[56] Hagberg M , Lundén A , Höglund J , Morrison DA , Waller KP , Wattrang E . 2008. Characterization of bovine lymphocytes stimulated in vitro by Dictyocaulus viviparus homogenate. Parasite Immunol 30:342–353. doi:10.1111/j.1365-3024.2008.01031.x 18444959

